# Variations in the Pulse of Different Arteries

**Published:** 1875-09

**Authors:** Moriz Benedict

**Affiliations:** Vienna


### VARIATIONS IN THE PULSE OF DIFFERENT ARTERIES.

By Dr. MORIZ BENEDICT, of Vienna.

(Translated from the "Wiener Med. Presse," No. 18, May 2, 1875, by Dr. H. Gradle.)

One of the secrets of the organic world is the differentiation of homogeneous cells, whence result, in the course of development, their different functions. But it is likewise astonishing, how the well-defined specific nature of the different elementary parts can be maintained permanently, bathed as they all are in a nutrient fluid uniform in composition. Of primary importance for this purpose is the difference in the nature of the filtering walls of the vessels, though we are not acquainted with the details of that arrangement.

A second factor, resulting in different exosmosis in different tissues under the same central pressure, is the specific mode of distribution of the blood vessels and lymphatics. Amongst anatomists much engaged with injections, it is a test of ability to recognize the source of such preparations, especially microscopical specimens. This relation is the most interesting in the brain, where there is an aggregation of nerve cells, almost identical chemically and histologically, and still endowed with such vastly different functions. Facts in toxicology, rather than micro-chemistry, demonstrate the great multiplicity in the chemical affinity of elements scarcely distinguishable by their anatomical characters.

Ecker has learned from his injected specimen that every part of the brain can be recognized by the vascular disposition peculiar to it. I will also call to mind at this place the peculiar relation of the large peripheral lymph-spaces in the arachnoid cavity of the brain, as well as the subadventitial lymph-spaces. During life there exists in the brain a normal accumulation of lymph (hyperlymphosis), the distension of the arachnoid cavity with fluid being dependent on the blood pressure. At the moment of death, the reimbibition into the tissues commences—a fact formerly known, upon which special stress has been laid lately by Schiff and Hitzig. These entire lymph-spaces, and in particular the subadventitial spaces, are adapted manifestly to the peculiar conditions in the brain, which, more than any other organ, may be exposed for weeks and even months to an unusual exertion, an unusual tissue-change, which without special protective arrangement would soon lead to alteration and destruction of the chemical individuality of the different elements. The unusual reservoirs and means of drainage for the nutrient fluid furnished in excess, and the excreta of the local process of nutrition, are, however, adapted to serve as protective arrangements; at the same time they are best calculated to prevent concussion of the brain.

Another peculiarity of the brain is the fact, that not only corresponding surfaces of both hemispheres are, as a rule, excited simultaneously, but that in the exercise of psychical functions elementary parts of the hemispheres widely distant are brought into synchronous excitation by irradiation. This relation is dependent on an intimate connection of the circulation; accordingly, we find not only communications between all arteries in the circle of Willis, but also, as Ecker, and, more lately, Heubner, have pointed out, do we see inosculations between the districts of the individual vessels in the pia mater, and hence, likewise, between the branches dipping into the cerebral convolutions.

But even these hydrostatic arrangements, as the result of which the central pressure is modified in the different tissues, would not suffice to maintain the chemical individuality of the tissues, were it not for another provision, capable of altering the velocity and tension of the blood. In artificial or functional irritation, as well as in irritation owing to contiguity of distant tissues, there must be some provision, by means of which the hæmadynamic conditions can be regulated according to the momentary requirements. The altered chemical condition of the irritated elements is one cause of an altered attraction; another factor is to be sought in the altered hæmadynamic mechanism.

*The local regulators of the acceleration or retardation of the blood velocity, or alteration of the blood pressure, are the vaso motor nerves. Through their influence the musculature of the vessels, which is but too imperfectly understood, attains the importance of a local heart.* This local heart is not capable—except in the hepatic artery—of augmenting by its activity the number of pulse-beats, nor can it probably reduce it, but it is able to bring about peculiar conditions of pressure and velocity, so that in the variety of association of activity of the separate nerves in a certain vascular district, we have not only an essential factor for the specific nutrition of the tissues, but also for an increase of nutrition during the time of exalted activity, and *vice versa.* To the same arrangement the trophic processes can be referred, and the time is probably not far distant at which we can trace a great part of the pathological processes to such disturbances of the local hearts, which take their origin in distant parts of the nervous system.

We know, at present, from the researches of Ludwig and Cyon, that an irritation starting in the heart can cause reflex dilatation of the vessels. On the other hand, we can hardly doubt but that the increase of central motive power on the occurrence of contraction of peripheral vessels, is also of reflex origin. Besides, we see in inflammatory fever, how intense local disturbances of circulation give rise to general disturbances of the vaso motor nerves; we see also, in the initial rigor, how the compensation of intense local dilatation of vessels is subject to physiological and *not* to hydrostatic laws. In fact, we can say, that the different local hearts are connected most intimately with each other and with the central heart by means of nervous bonds, the relations of which have been but imperfectly demonstrated by the means of anatomy and physiology.

It would be of the greatest importance to study in general the physiology of the terminal arteries, those branches regulating the nutrition of the organs; this, however, can be but imperfectly accomplished. But fortunately we are able to observe the distinct disturbances occurring in a terminal artery, *as a rebound, an echo, so to speak, in the larger arteries*, though this idea and its resulting observations have not as yet been carried out by physicians. Even in physiology we still meet with the methodic error of referring changes of tension caused by experimental stimulation or paralysis of nerves, which are observed in one region—to the entire vascular system. But in fact we can observe such local alterations in certain vascular districts, on the large vessels the pulse of which is perceptible to the finger, and some noticeable observations have induced me for some years to feel the pulse simultaneously in different arteries.

The first two observations are the following:

A lady of about 40 years of age came under my observation, with all the symptoms of an obstructed circulation in the arm. The hand was pale, cold, insensible, and at the same time painful. No pulse could be felt anywhere along the arm. The pulse of the subclavian artery was enormously full, so that at first sight the existence of an aneurism appeared probable. The history showed that this was not a congenital state, but only of eight days duration. Embolism and thrombosis could be excluded on account of the relative want of importance of the symptoms, and the idea occurred to me, that there existed a vascular spasm, not sufficiently intense to stop the circulation, but sufficient to render the pulse imperceptible. The attempt to remove this state of the vaso motor nerves by belladonna and galvanization did not succeed; but after a few days the normal condition returned.

The subject of another observation was a young girl, complaining of headache and palpitation of the heart. On examination, I noticed that both carotid and both radial arteries showed an unusual, though equally full, pulse. Besides, all symptoms of an insufficiency of the bicuspid valve were present. The unusual fullness and hardness of the pulse in the region of the ascending aorta gave me the idea of examining the pulse in the region of the descending aorta, where I found that no pulse could at all be perceived. Recollecting my former case, the thought occurred to me that there might be in this case also a spasm of the arteries in the latter region; but a congenital obliteration in the descending part of the arch of the aorta was first to be excluded. All appearances of a collateral circulation were wanting. I insisted on a consultation with a celebrated authority on diseases of the circulatory organs. Shortly afterwards the pulse reappeared in the lower extremities, and the abnormal characters of the carotid and radial pulse disappeared, and even the apparent cardiac lesion was said to have vanished subsequently.

I made some other very striking observations on two other patients. One of them suffered for a long time from clonic spasm, consisting of flexion of the carpus, which involved occasionally also the elbow-joint, persisting for hours, and accompanied by diminished sensibility of the fingers; its duration was shortened by warmth. The attacks occurred repeatedly during the day, and would sometimes last during a whole day. After severe attacks a sense of constriction was felt in the chest, the radial pulse was not perceptible on the right side, while on the left side it was very small and irregular. One day, when the patient had just passed out of an attack, an attempt was made to enlarge the pulse by nitrite of amyl.* In this I succeeded very promptly, and after the experiment had been repeated twice, both on palpation and auscultation a momentary arrest of the heart was observed, while a deep bronchial respiration was heard. After a few seconds the heart sounds became audible again, and after the lapse of ten minutes the cardiac impulse was very powerful. The radial pulse remained full for some time. (Still the attack continued for some hours, notwithstanding the experiment.) This experiment shows also what extent the vascular spasm can attain. A permanent enlargement of the pulse was only obtained by galvanization of the sympathetic; whether, however, a cure was effected, I could not learn.

A second instance was the case of an epileptic man, whose attack was always preceded by a premonitory vaso motor symptom in the shape of circumscribed redness of the left cheek.† I saw the man one day at this period, and an examination showed the pulse filiform in the carotid as well as the subclavian and radial arteries and the lower extremities. By strong galvanization of the sympathetic I succeeded in rendering the pulse fuller, and the attack was prevented.

No less striking are the appearances in *tic douloureux*, where either the pulse of the carotid artery is full during the attack or on the hand very small. That the carotid artery can be dilated like an aneurism by the expansion of the places of origin of the smaller arteries, I have witnessed both in tic douloureux (vide case 87 of the "Nervenpathologie") and hydrophobia. In the former

* Riegel has lately used this remedy to dilate the arteries in lead poisoning.

† I will here insist that an epileptic attack rarely occurs without vaso motor or psychical prodromata.

case the abnormal distension of the vessel disappeared with the affection, and in the latter case no anomaly was found in the cadaver.

Spasms of vessels or pareses in limited vascular districts are also a common symptom in hysteria.

I attach special importance to this method in diseases of the brain. Under normal circumstances a difference is often noted between the pulse of both carotids, and, as a rule, the radial pulse corresponding to the large carotid artery is in that case the smaller. In hemiplegia and cerebral disease, I have repeatedly observed, that on one side (in the former case the paralyzed side) both the pulse of the common carotid and of the arteries of the arm was small and filiform. Such a condition denotes a profound disturbance of circulation by dilatation in numerous vascular districts, and my idea to prognosticate from this condition the danger of apoplexy has in several instances been unhappily confirmed.

In Basedow's disease the pulse is usually large in both carotids and correspondingly small in both radial arteries. I am not at present enabled to perfect these observations on a larger scale; I can, therefore, merely recommend them most urgently to my colleagues. We can expect from this method not only the discovery of special facts, but even a further illumination of the causes of trophic processes and an increased accuracy of prognosis. Especially in local inflammations in the extremities can the observation of the local arterial tension be of interest.

---

Fleetwood Churchill, author of "Churchill's Obstetrics," and kindred works, has lately retired from practice. His practice for many years of late has been confined wholly to obstetric and gynecologic consultations.

